# Female Endurance Runners Have a Healthier Diet than Males—Results from the NURMI Study (Step 2)

**DOI:** 10.3390/nu14132590

**Published:** 2022-06-22

**Authors:** Mohamad Motevalli, Karl-Heinz Wagner, Claus Leitzmann, Derrick Tanous, Gerold Wirnitzer, Beat Knechtle, Katharina Wirnitzer

**Affiliations:** 1 Department of Sport Science, Leopold-Franzens University of Innsbruck, 6020 Innsbruck, Austria; seyed.motevalli-anbarani@student.uibk.ac.at (M.M.); derrick.tanous@student.uibk.ac.at (D.T.); 2 Department of Subject Didactics and Educational Research and Development, University College of Teacher Education Tyrol, 6010 Innsbruck, Austria; 3 Department of Nutritional Sciences, University of Vienna, 1090 Vienna, Austria; karl-heinz.wagner@univie.ac.at; 4 Institute of Nutrition, University of Gießen, 35390 Gießen, Germany; claus@leitzmann-giessen.de; 5 AdventureV & Change2V, 6135 Stans, Austria; gerold@wirnitzer.at; 6 Institute of Primary Care, University of Zurich, 8091 Zurich, Switzerland; beat.knechtle@hispeed.ch; 7 Medbase St. Gallen Am Vadianplatz, 9001 St. Gallen, Switzerland; 8 Research Center Medical Humanities, Leopold-Franzens University of Innsbruck, 6020 Innsbruck, Austria

**Keywords:** sex, gender, nutrition, dietary assessment, food frequency, protein, fruit, vegetables, distance running, half-marathon, marathon

## Abstract

Sex has been recognized to be an important indicator of physiological, psychological, and nutritional characteristics among endurance athletes. However, there are limited data addressing sex-based differences in dietary behaviors of distance runners. The aim of the present study is to explore the sex-specific differences in dietary intake of female and male distance runners competing at >10-km distances. From the initial number of 317 participants, 211 endurance runners (121 females and 90 males) were selected as the final sample after a multi-level data clearance. Participants were classified to race distance (10-km, half-marathon, marathon/ultra-marathon) and type of diet (omnivorous, vegetarian, vegan) subgroups. An online survey was conducted to collect data on sociodemographic information and dietary intake (using a comprehensive food frequency questionnaire with 53 food groups categorized in 14 basic and three umbrella food clusters). Compared to male runners, female runners had a significantly greater intake in four food clusters, including “beans and seeds”, “fruit and vegetables”, “dairy alternatives”, and “water”. Males reported higher intakes of seven food clusters, including “meat”, “fish”, “eggs”, “oils”, “grains”, “alcohol”, and “processed foods”. Generally, it can be suggested that female runners have a tendency to consume healthier foods than males. The predominance of females with healthy dietary behavior can be potentially linked to the well-known differences between females and males in health attitudes and lifestyle patterns.

## 1. Introduction

The importance of sex-related comparison in sports nutrition topics has been widely discussed over the past decade [1]. It is well-established that the nutritional requirements of athletes are potentially affected by physical and physiological differences between males and females [2,3]. These sex-based differences seem to be more predominant in ultra-endurance athletes who are recommended to pay superior attention to their specific nutritional needs due to the prolonged training/racing activities [4,5].

Sex differences in endurance performance are not limited to the menstrual cycle that causes unfavorable effects on training procedures in female athletes (mainly due to the associated challenges and anemia rather than hormonal fluctuations) [6,7]. Evidence shows that females have a lower oxygen-carrying capacity (due to fewer erythrocytes and hemoglobin levels) than males, which can affect their endurance performance negatively [8]. In addition, females are more susceptible to developing thyroid disorders compared to males [9] resulting in performance-limiting outcomes, including fatigue [10]. However, males seem to be more prone to cardiovascular abnormalities as it has been shown that cardiac death and coronary heart disease are more prevalent in males than females [11,12], which increases the likelihood of unfavorable health- and performance-related consequences. Considering the fact that male athletes are characterized as being more influenced by risky behaviors such as performance-enhancing substance abuse [13,14], their cardiovascular health is of greater concern. Research indicates that in muscle metabolism pathways during endurance activities, females have a higher capacity to utilize muscle lipids as fuel, and males rely more on muscle and liver glycogen resources [15,16]. To achieve an optimal level of endurance performance, however, females may need further training adaptations compared to males [17,18] due to the basic sex-specific physical differences (e.g., body mass, muscle mass, and fat mass) [6,19].

Nutritional requirements and patterns may also be affected by sex, whether dependent or independent of the mentioned physical and physiological differences between males and females. It has been shown that female athletes have a greater prevalence of unintentional caloric imbalance than males in order to reach and maintain the appropriate body composition required for an optimized level of endurance performance [6,18,19]. Females have also been reported to be generally more health conscious than males, which also can be associated with their attitudes towards food choice, including a greater intake of fruits, vegetables, and whole foods [20]. In contrast, it has been shown that males are more motivated to increase physical activity in their daily routines rather than modifying their nutritional habits [21]. Generally, the various health- and lifestyle-related beliefs between females and males have been predicted to be responsible for up to 50% of sex-specific dietary choices [20].

Dietary assessment is a crucial part of sports nutrition practice, which helps identify nutritional inadequacy (that commonly occurs following restrictive diets) and optimize dietary strategies for improving performance and health. Nutritional concerns, particularly energy deficiency, are more critical in both male and female long-distance runners compared to those who run in shorter races [18,22]. Likewise, nutritional requirements are positively associated with increasing intensity, duration, and frequency of running/training sessions [18,23]. Data show that typical daily foods may not fulfill the nutritional needs of endurance runners to support their physiological requirements [22,24]. This concern is more serious for endurance athletes who follow unbalanced and/or inappropriately-planned diets, which has been shown to occur in all diet types (e.g., omnivorous or plant-based diets) [25,26,27]. It has been reported that even ultra-endurance events can be completed successfully without any health-related consequences by athletes who consume only plant-based foods [28,29]. This finding supports that by following the well-recognized dietary guidelines, appropriately planned plant-based diets can maintain the health of long-distance runners [28,29].

Regardless of the well-established sex differences in physical, physiological, and nutritional characteristics of general populations [30], there is limited evidence comparing dietary intake between male and female endurance athletes, particularly distance runners. Despite the advancement of knowledge in illustrating sex-based differences, the majority of sports nutrition topics have a paucity of female-specific examinations, resulting in the misapplication of many scientific conclusions for female athletes [31]. Available studies regarding the nutrient requirements of endurance athletes [32,33,34] are not consistent in covering all sex-based differences, or they did not distinguish race distance and diet type of female and male endurance runners [35,36]. Therefore, the present study was conducted to investigate and compare the dietary intake of female and male distance runners across different subgroups of diet type and race distance. It was hypothesized that female runners have a dietary intake more advantageous to health.

## 2. Materials and Methods

### 2.1. Study Design and Ethical Approval

The present study is a part of the Nutrition and Running High Mileage (NURMI) Study Step 2. The study protocol [37] was approved by the ethics board of St. Gallen, Switzerland (EKSG 14/145; 6 May 2015) with the trial registration number ISRCTN73074080. The methods of the “NURMI Study Step 2” have been previously described in detail [38,39].

### 2.2. Participants and Experimental Approach

Endurance runners were mainly recruited from Austria, Germany, and Switzerland and were contacted via social media, websites of organizers of marathon events, online running communities, email lists, and runners’ magazines, as well as via additional/other multi-channel recruitments and through personal contacts. Participants were asked to complete an online survey within the “NURMI Study Step 2”, which was available in German and English (https://www.nurmi-study.com/en (accessed on 10 May 2022)). Participants were provided with a written description of the procedures and gave their informed consent before completing the questionnaire. The following inclusion criteria were initially required for successful participation in the “NURMI Study Step 2”: (1) written informed consent; (2) at least 18 years of age; (3) questionnaire Step 2 completed; (4) successful participation in a running event of at least half-marathon distance in the past two years.

Female and male participants were further categorized according to race distance and kind of diet. Race distance subgroups were half-marathon and (ultra-)marathon (data were pooled since the marathon distance is included in an ultra-marathon); the shortest and longest ultra-marathon distances reported were 50 km and 160 km, respectively. However, a total number of 74 runners who completed the 10-km distance, but had not successfully participated in either a half-marathon or a marathon, also provided accurate and useable answers similar to runners competing over half-marathon or higher. In order to avoid an irreversible loss of these valuable data sets, those who met the inclusion criteria (1) to (3) were kept as additional race distance subgroup. Dietary subgroups were omnivorous (or Western diet, with no restriction on any food items), vegetarian (devoid of all flesh foods, including fish and shellfish, but including eggs and/or dairy products), and vegan diet (devoid of all foods from animal sources, including honey) [40,41] with a minimum of 6-month adherence to the self-reported diet types.

### 2.3. Data Clearance

**Figure 1:**
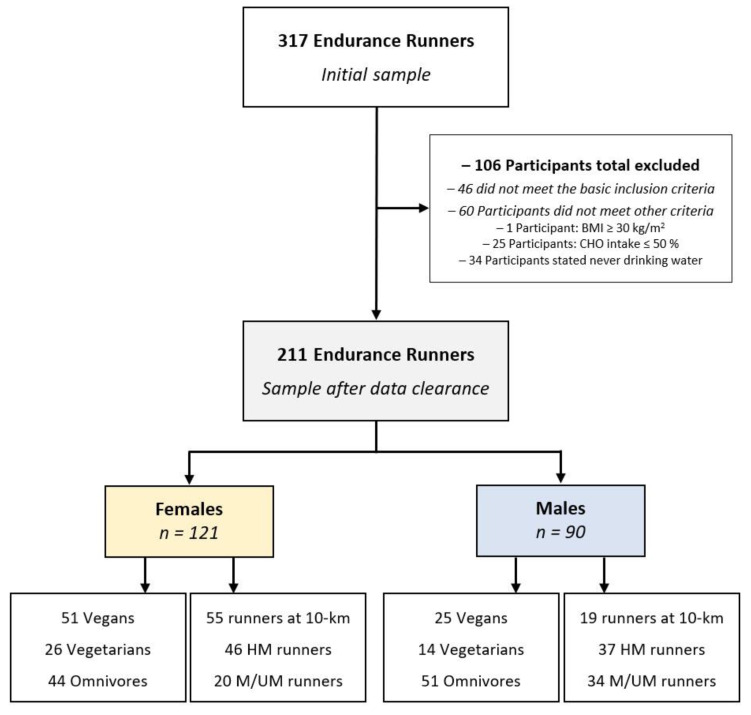
Participants’ enrollment and classifications by sex.

From the initial number of 317 endurance runners, a total of 106 participants were excluded from the data analysis. Of these, 46 participants did not meet the basic inclusion criteria. In order to control for a minimal status of health linked to a minimum level of fitness and to further enhance the reliability of data sets, the Body Mass Index (BMI) approach following the World Health Organization (WHO) standards [42,43] was applied. On this basis, one participant with a BMI ≥ 30 kg/m^2^ was excluded from the data analysis since first other health-protective and/or weight loss strategies other than running are necessary to safely reduce body weight. Further, as a result of the specific exclusion criteria for the present study, an additional number of 25 runners were identified and excluded for consuming ≤50% carbohydrates of their total dietary intake (which is lower than the minimum level recommended for maintaining a health-performance association [25,44,45]). Moreover, 34 participants with conflicting statements on water intake (e.g., stated never drinking water) were excluded from the analysis to avoid conflicting data on dietary intake [44]. In addition, a total of 24 runners (11%) had to be shifted to other dietary subgroups: 4 vegan runners: respectively 2 to omnivores and 2 to vegetarian samples; and 20 (9%) vegetarian runners had to be shifted to the omnivores subsample. However, 89% (*n* = 187) of the recreational runners correctly assessed their kind of diet. As the final sample, 211 runners (121 women and 90 men) with complete data sets were included for statistical analysis. Figure 1 shows the participants’ enrollment and classifications within the present study.

### 2.4. Measures and Statistical Modelling

Based on the food frequency questionnaire (FFQ) of the “German Health Interview and Examination Survey for Adults (DEGS)” (DEGS-FFQ; with friendly permission of the Robert Koch Institute, Berlin, Germany) [46,47], participants were asked to report their regular food intake based on the consumption frequency (single-choice out of 11 options ranging from “never” to “5 times a day”) and quantity of a broad variety of specific dietary items (single-choice from various options depending on the food group) particularly in the past four weeks, including meals eaten while out, i.e., in restaurants, canteens, at friends’ houses, etc.

Based on the 53 food groups of the DEGS-FFQ and following the Nova classification system of the Food and Agriculture Organization (FAO, Rome, Italy) [48,49,50,51], subgroups of foods were categorized with the corresponding questions pooled for a total of 17 food clusters in order to perform quantitative and qualitative data analyses (Table 1). Self-reported data, including sociodemographic information, motive(s) for diet type adherence, and pooled food frequency, were linked to sex-based groups.

### 2.5. Statistical Analysis

The statistical software R version 4.1.1 (10 August 2021) Core Team 2018 (R Foundation for Statistical Computing, Vienna, Austria) was used to perform all statistical analyses. Exploratory analysis was done by descriptive statistics: mean values and standard deviation (SD), median and interquartile range (IQR). Chi-square tests (χ^2^, nominal scale) were conducted to examine the association of sex with nationality, marital status, academic qualification, diet type, race distance, and dietary motives. Kruskal–Wallis tests (ordinal and metric scale) were approximated by using the t or F distributions or using ordinary least squares and standard errors (SE) with R^2^ to test the association of sex with age, body weight, height, and BMI. Food cluster as the latent variable was derived by 53 manifest parameters (assessing how often and how much consumption of specific dietary items). In order to scale the food consumption displayed by measures, items, and clusters, a heuristic index (as a new composite variable) ranging from 0 to 100 was defined (equivalence in all items; FFQ was calculated by multiplying the reports of both questions, and dividing by the maximum then). A linear regression model was used to examine significant differences in the intake of specific food clusters by sex and age. The assumptions of the regression analysis have been verified by inspection of graphs of residuals. Differences in respective food clusters between females and males are displayed as effect plots (95% confidence interval). The level of statistical significance was set at *p* ≤ 0.05.

## 3. Results

From a total of 211 runners (including 121 females and 90 males) with a median age of 38 (IQR 18) years, there were 74 runners of 10-km, 83 half marathoners, and 54 marathoners/ultramarathoners based on race distance, and 95 omnivores, 40 vegetarians, and 76 vegans based on kind of diet. The majority of endurance runners (96%) were from German-speaking countries (i.e., Germany, Austria, and Switzerland), while 4% of participants were from other countries worldwide.

Descriptive analysis showed significant differences between females and males in age (*p* = 0.023), where males with a median age of 42 (IQR 17) years were older than females with a median age of 37 (IQR 15) years, and BMI (*p* < 0.001), where males had a higher BMI (22.91 kg/m^2^, IQR 2.86) compared to females (20.94 kg/m^2^, IQR 3.05). No significant difference (*p* > 0.05) was found between male and female runners in academic qualification or marital status. There was a significant sex-based difference in race distance (*p* < 0.001), where the majority of 10-km runners and half marathoners were female, and most marathon/ultramarathon runners were male. A significant sex-based difference was detected in diet type (*p* = 0.013), as vegetarian and vegan diets were more common in females and omnivorous were more prevalent in male runners. While endurance runners reported mostly “health & wellbeing” (by 85%) as the main reason/motive to adhere to their self-reported diet types, “social aspects” was the only motive with a significant difference between females and males (41% vs. 65%, respectively; *p* = 0.010). Table 2 shows the sex-based differences in sociodemographic characteristics of the participants.

Significant differences between female and male participants were found in the consumption of 11 out of 17 food clusters (*p* < 0.05). Compared to males, female runners reported a greater intake of four food clusters including beans and seeds (*p* = 0.008), fruit and vegetables (*p* < 0.001), dairy alternatives (*p* = 0.012), and water (*p* = 0.002). In contrast, males had a higher intake of seven food clusters including grains (*p* < 0.001), meat (*p* < 0.001), fish (*p* = 0.033), eggs (*p* = 0.041), oils (*p* = 0.033), alcohol (*p* < 0.001), and processed foods (*p* = 0.001). There was no significant difference between female and male runners in the consumption of six food clusters, including dairy (*p* = 0.159), meat alternatives (*p* = 0.488), snacks (*p* = 0.086), beverages (*p* = 0.350), protein (*p* = 0.599), and free/added sugar (*p* = 0.212). Table 3 displays the sex-based differences in intake of 17 food clusters and the subset items.

**Figure 2:**
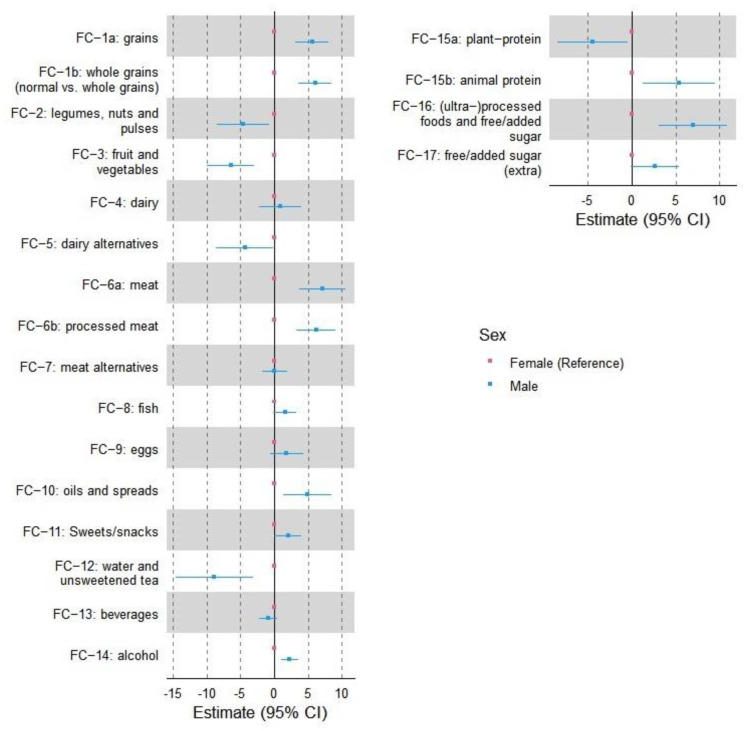
Forest plots with 95% confidence interval to display sex-based differences in basic (the left column) and umbrella (the right column) food clusters. Females are considered the reference, and the differences are shown based on the variations of males from females. FC—food clusters.

Figure 2 displays the 95% confidence interval to show sex-related differences in food clusters in runners. The food clusters with more than 5% difference between males and females include “grains” (both subclusters: refined and whole grains), “meat” (both subclusters: unprocessed and processed meat), “animal protein”, “processed foods & free/added sugar”, “fruit and vegetable”, and “water and unsweetened tea”, where males had a higher consumption compared to the opposite sex in the first four clusters and female in the two latter clusters.

Further details regarding the regression results, including *p*-values, are presented in Table 4. Age was a significant predictor for consumption of the cluster “fruit and vegetables” (*p* = 0.010), with a marginal (but not significant) association with the two clusters “eggs” (*p* = 0.058) and “plant protein” (*p* = 0.056).

## 4. Discussion

The present study investigated and compared female and male endurance runners in dietary intake (differentiated by 14 basic clusters and 3 umbrella clusters of food frequency). The most important findings were that (1) females had a significantly higher intake of four food clusters (i.e., “beans and seeds”, “fruit and vegetables”, “dairy alternatives”, and “water”) than males; (2) males had a significantly greater intake of seven food clusters (i.e., “grains”, “meat”, “fish”, “eggs”, “oils”, “alcohol”, and “processed foods”) than females; (3) no significant sex-based difference was observed in the consumption of six food clusters (i.e., “dairy”, “meat alternatives”, “snacks”, “beverages”, “protein”, “free/added sugar”); (4) sex has been found to be a significant predictor for consumption of the majority of food groups; (5) except for “fruit and vegetables” age failed to be a significant predictor of the food groups. As another main outcome, the hypothesis of the present study i.e., “female runners having a more advantageous dietary intake regarding a healthy lifestyle“, was verified.

The purpose of the dietary assessment was to identify nutritional inadequacy in order to optimize health-related approaches in general populations and develop individualized dietary strategies for improving the health and performance of athletes [52,53]. Overall, the most common dietary assessment methods include a dietary record, 24-h dietary recalls, in-depth interviews, and the food frequency questionnaire [52,53,54]. Evidence has shown that food records, dietary recalls, and detailed interviews are time-consuming and challenging to conduct precisely in athletes [55,56]. On the other hand, food frequency questionnaires have been reported to be a simple, fast, and low-cost method with less burden on participants compared to other methods [57]. Hence, food frequency questionnaires can be the most appropriate survey method to assess the dietary intake of athletes [57,58]. Athletes in general–but particularly those who follow restrictive and unbalanced diets–are at higher risk for low energy intake than sedentary people if their diet is not planned appropriately [25,59]. Considering the importance of diet for health status and athletic performance, it is crucial that the first and most important step in any sports nutrition practice is to assess and monitor the dietary intake/status of athletes [56].

In line with the findings from the present study, it has been reported that sex is an important predictor of dietary choices, which mainly originates from different health and lifestyle beliefs between males and females [20]. According to the literature, the influence of sex on dietary intake is not limited only to runners [60,61] but has also been documented in the general population [62,63]. Reports from national dietary investigations on general populations of D-A-CH countries (including Germany, Austria, and Switzerland; home of the majority of participants) also show that sex is a remarkable contributor to dietary intake/patterns [64,65,66,67]. Dietary-related sex differences in endurance runners, however, cannot only be attributed to the patterns of supplement intake, as previously reported by the “NURMI Study” [68].

### 4.1. Fluid and Alcohol

In the present study, data on hydration habits revealed that sex seems to be an influencing variable in the consumption of water (with a predominance of females) but not beverages. Comparable results from an investigation of recreational runners showed a significant sex-based difference in the type of fluid intake, where female runners consumed more water, coffee, and tea, and males more sweet beverages or alcoholic drinks [60]. National dietary reports for the German population also indicated a greater consumption of water, coffee, and tea in females than males [67]. Consistently, male runners in the present study reported nearly a two-fold consumption of alcohol compared to females. While data from the Austrian general population show that males had a 3-times greater intake of alcohol than females [65], this ratio was 2:1 in a similar investigation in Switzerland [66]. According to the dietary recommendations of D-A-CH nutrition organizations, the sex-based differences in the maximum tolerable alcohol intake is also two-fold (i.e., max. 10 g/day for healthy females and max. 20 g/day for healthy males) [69]. Generally, male athletes are at a higher risk of binge drinking than females [60,70].

### 4.2. Carbohydrate Foods

The consumption of grains (both refined and whole grains) was higher in males than females in the present study. This finding is inconsistent with the results from the national German report, where females had a higher intake of grains and cereals [67]. Assuming an equal ability of females and males to store and utilize carbohydrates [71], the present finding might be associated with the increased portion of females in 10-km and males in M/UM subgroups. In this regard, it has been reported that sex difference in carbohydrate intake is likely to disappear when the data is adjusted to training volume [61]. Consistent with the present findings, results from a comparable investigation show that female distance athletes tend to consume fewer carbohydrates than males [72]. Grains are not the only source of carbohydrates since other food clusters (e.g., “fruit and vegetables” and “beans and seeds”) also contribute to the carbohydrate supply. Female runners in the present study reported a higher intake of both the clusters “fruit and vegetables” and “beans and seeds” than males. Consistently, it has been documented that females are more eager than males to consume fruits and vegetables [20], and this food cluster showed the highest contrast between the dietary patterns of females and males [73]. The significant predominance of females in the consumption of fruit and vegetables has also been shown by German [64,67], Swiss [66], and Austrian [65] studies on general populations. However, it was unanimously found that the majority of both males and females do not reach the recommendation of five portions of fruits and vegetables per day. Regarding dietary attitudes, while females more frequently than males indicated that vegetables are the major component of a healthy diet, they expressed that the consumption of carbohydrates should be decreased [68]. This finding may be linked to the heightened concerns about body image among females in general populations, and especially female athletes [62,74].

### 4.3. Protein and Fat-Based Foods

Research has shown that male athletes have a generally higher protein intake than recommended [4,75]. This outcome, however, is not consistent with the nutritional recommendations indicating the greater need for baseline protein intake for female endurance athletes due to their higher rate of protein oxidation than males [76,77]. Male runners in the present study reported a generally greater intake of animal protein foods (meat, fish, eggs) than females; however, no sex-based difference was observed in the consumption of dairy products and meat alternatives. The predominance of males in the consumption of meat has been periodically shown in national studies on general populations of Germany [67], Austria [65], and Switzerland [66]. Although animal sources derive approximately 75% of the general protein supply in athletes [78], it has been reported that both male and female marathoners consume a higher portion of plant-based proteins than other athletes and the general population [75,79]. Dietary shifts toward a lower intake of animal sources and more plant foods can result in a lower intake of processed meat (including fast foods) and high-fat foods [80] and consequently improve health and performance [25]. The present findings also indicate a greater consumption of oils and processed foods by male runners. While similar investigations on athletes [3] and general populations [61] support the present findings, the most reasonable justification that has been reported is the lower ability and time of males in preparation of meals, which leads them to consume convenient/fast food and restaurant meals [3].

### 4.4. Health Insights in Food Intake

In general, the present findings show that female runners have a tendency towards a healthier dietary intake pattern than their male counterparts. It has been reported that female athletes mainly prefer to consume dietary sources containing more micronutrient density to fulfill their health-related concerns [20,30,81], whereas male athletes seem more interested in consuming macronutrients, especially from protein sources, aiming to maintain and improve muscle mass and strength. It has also been found that the prevalence of consuming high-fiber meals (as an indicator of a healthy diet) is considerably higher in females than males [20].

The general higher intake of healthier food clusters by female runners appears to be linked to the higher level of females’ health consciousness compared to males [20,82]. Regardless of sex, previous findings from the NURMI project show that runners who follow a vegan diet had a higher level of health consciousness, mainly due to their more beneficial choice of dietary items compared to non-vegan runners [38]. Such sex-based differences in health consciousness and dietary behaviors can also be associated with the well-documented fact that females are generally more interested in diet and health, while males consider physical activity as the main part of a healthy lifestyle [30]. However, it is necessary to consider that regular physical activity, independent of sex, alters the attempts toward a healthier dietary pattern in order to gain further outcomes [83]. As a general fact in sport science, different nutritional requirements of athletes competing in different types of sports should also be considered a potential factor to justify dietary contradictions [36]. Educational level and, more importantly, specific knowledge about nutrition and sport sciences may also be associated with health behaviors, particularly adhering to a healthier diet [84]. In terms of academic qualification, however, there was no significant difference between female and male participants in the present study. The unbalanced distribution of race distance and diet type subgroups across male and female groups may partially contribute to the finding on sex-based dietary differences.

Unlike sex, age was not a significant predictor for consumption of the majority of food groups except for one food cluster (i.e., fruit and vegetables). While the null effect of age on general dietary intake can be linked to the fact that male runners were significantly older than female runners, data from dietary studies on general populations indicate that age can be a moderate indicator of dietary patterns [30,73]. It should be considered that most participants in the present study were recreational runners. Evidence indicates that performance level, defined as the term professionalism, can be a key indicator of precise and personally tailored dietary intake and strategies for training and racing independent of age [85,86]. In this regard, the literature reports that the major motives of recreational athletes to take part in sport events are health and/or hobby [87,88], while professional athletes are mainly motivated by performance and competition-related aspects [89].

### 4.5. Limitations and Strengths

Some limitations in the present investigation should be mentioned. The study was conducted following a cross-sectional design producing self-reported findings; therefore, caution should be taken by interpreting the results. However, several control questions were implemented in different parts of the survey to minimize validity bias and control for contradictory data, and accordingly, participants’ statements were checked for congruency and meaningfulness. The unbalanced distribution of diet type and race distance subgroups among male and female groups (Figure 1) may also be considered as another limitation affecting the sex-based findings and interpretations. Moreover, as a potential selection bias, about half of the endurance runners in the present study stated adhering to a vegan or vegetarian diet, which is markedly higher than the prevalence in general populations. Finally, despite the well-approved validity of a FFQ as a practical method to assess dietary intake and patterns [56,57], especially for athletic populations [57,58], this method seems unsuited to provide details about the macro- and micro-nutrient status of the athletes (on which a considerable number of nutritional recommendations are based on).

However, the findings contribute valuable and novel data to current scientific knowledge regarding the sex-related patterns of dietary intake among recreational endurance runners categorized across different subgroups of diet types and race distance. Although the present study opens a direction for future interventional studies on athletic populations, future research with larger and more differentiated samples of distance runners will assist in providing comparable data for a better understanding of the dietary patterns of female and male runners.

Finally, the results from the present study will also provide a window into the targeted sex-specific approaches to precisely tailor and personalize the dietary needs and nutritional requirements of male and female distance runners. Endurance runners, their coaches, and sports nutrition specialists can benefit from the results when designing and applying nutritional strategies for long-term adherence to training and competition.

## 5. Conclusions

The sex-based comparison of endurance runners showed that there are remarkable differences between females and males in their dietary intake (assessed by a food frequency questionnaire), supporting the fact that female runners tend to consume healthier foods. While physiological differences between females and males can play a key role in many sex-based nutritional and behavioral variances, it seems that health-oriented attitudes and lifestyle of females can be considered the most reasonable justification for the present findings. However, there is an obvious necessity to design more detailed interventions using further analyses of interacting factors to improve the knowledge of sex differences in dietary choices of endurance athletes and, consequently, to support sports dietitians, nutritionists, and coaches to provide more precise and personalized recommendations. In general, nutrition education, training opportunities, and sports nutrition counseling to expand a runner’s personalized knowledge about health and sports discipline-specific behaviors can be recommended practically to improve the healthy runner lifestyle, including nutritional competencies (e.g., healthy ingredients, nutrients as well as requirements, and foods) in matching the higher exercise-induced demands for active males and females alike.

## Figures and Tables

**Table 1:** Modeling of the Clusters for Food Frequency (Basic Nutrition and Consumption Cluster 1 to 14; Umbrella Cluster for Preparation Cluster 15 to 17).

Basic Food Clusters		
Cluster 1	Grains a—grains b—whole grains	cornflakes; white bread; white pasta muesli; wholegrain; mixed bread; wholegrain pasta; wholegrain rice; other grains
Cluster 2	Legumes, nuts, and pulses	pulses; nuts and seeds; legumes
Cluster 3	Fruit and vegetables	vegetable juice; fruit; vegetables
Cluster 4	Dairy products	milk; cheese; yoghurt
Cluster 5	Dairy alternatives	milk alternatives
Cluster 6	Meat a—meat b—processed meat	chicken; beef; pork; deer fried nuggets; hamburger; sausage; kebab; pork; processed meat
Cluster 7	Meat alternatives	tofu; seitan; tempeh; etc.
Cluster 8	Fish, shellfish, and seafood	
Cluster 9	Eggs	
Cluster 10	Oils and spreads	butter; margarine; oils
Cluster 11	Sweets and snacks	sweets; snacks; salty snacks
Cluster 12	Water and unsweetened tea	
Cluster 13	Beverages	
Cluster 14	Alcohol	
**Preparation/Umbrella Clusters**		
Cluster 15	Protein a—plant protein b—animal protein	legumes and beans; vegetables; grains (couscous, quinoa); dairy alternatives (e.g., soy products); meat alternatives dairy products; eggs; meat and processed meat products; fish, seafood, and shellfish
Cluster 16	(Ultra-)processed foods and free/added sugar	sugary carbonated drinks; kcal reduced/artificially sweetened drinks; fruit juice; free sugar in tea; free sugar in coffee; cereals; sweet and savory spreads; margarine; pasta; sweets, cakes, and biscuits; salty snacks, butter; processed meat; processed plant products
Cluster 17	Free/added sugar	Sweet spread; sugary carbonated drinks; fruit juice; free sugar in tea; free sugar in coffee; cereals; sweets, cakes, and biscuits

**Table 2:** Sociodemographic characteristics of female and male runners.

		Total *n* = 211	Females *n* = 121	Males *n* = 90	Statistics
Age (years)		38 (IQR 18)	37 (IQR 15)	42 (IQR 17)	F_(1, 209)_ = 5.26; *p* = 0.023
Body Weight (kg)		65.0 (IQR 14.1)	59.8 (IQR 10.6)	73.6 (IQR 12.3)	F_(1, 209)_ = 189.68; *p* < 0.001
Height (m)		1.7 (IQR 0.1)	1.7 (IQR 0.1)	1.8 (IQR 0.1)	F_(1, 209)_ = 191.83; *p* < 0.001
BMI (kg/m^2^)		21.72 (IQR 3.40)	20.94 (IQR 3.05)	22.91 (IQR 2.86)	F_(1, 209)_ = 33.21; *p* < 0.001
Academic Qualification	Upper Secondary/Technical A Levels or Equivalent University/Higher Degree No Answer	33% (69) 23% (49) 34% (72) 9% (21)	30% (36) 23% (28) 36% (43) 12% (14)	37% (33) 23% (21) 32% (29) 8% (7)	χ^2^_(3)_ = 2.14; *p* = 0.709
Marital Status	Divorced/Separated Married/Partner Single	5% (11) 68% (143) 27% (57)	7% (8) 61% (74) 32% (39)	3% (3) 77% (69) 20% (18)	χ^2^_(2)_ = 5.75; *p* = 0.056
Country of Residence	Austria Germany Switzerland Other Countries	17% (36) 74% (156) 5% (11) 4% (8)	10% (12) 80% (97) 5% (6) 5% (6)	27% (24) 66% (59) 6% (5) 2% (2)	χ^2^_(3)_ = 11.03; *p* = 0.012
Race Distance	10-km HM M/UM	35% (74) 39% (83) 26% (54)	45% (55) 38% (46) 17% (20)	21% (19) 41% (37) 38% (34)	χ^2^_(2)_ = 17.95; *p* < 0.001
Diet Type	Omnivorous Vegetarian Vegan	45% (95) 19% (40) 36% (76)	36% (44) 21% (26) 42% (51)	57% (51) 16% (14) 28% (25)	χ^2^_(2)_ = 8.64; *p* = 0.013

Note. IQR—Interquartile range. BMI—body mass index. km—kilometers. HM—half-marathon. M/UM—marathon/ultra-marathon. Statistical methods: Kruskal–Wallis tests (represented by median and IQR data) and Chi-square tests (represented by prevalence data).

**Table 3:** Differences between female and male runners in food clusters and items.

	Females	Males	Statistics
	*n* = 121	*n* = 90	
**Part A—Basic clusters**			
FC—1 (Total of grains)	15.43 ± 7.86	21.90 ± 8.16	F_(1, 209)_ = 36.40; *p* < 0.001
FC—1a (Total of refined grains)	9.99 ± 8.14	15.54 ± 9.57	F_(1, 209)_ = 19.64; *p* < 0.001
Cornflakes	1.60 ± 3.57	1.44 ± 4.99	F_(1, 209)_ = 2.34; *p* = 0.127
White bread	6.07 ± 6.35	10.36 ± 9.18	F_(1, 209)_ = 12.03; *p* = 0.001
White pasta	8.81 ± 8.48	13.84 ± 9.42	F_(1, 209)_ = 16.03; *p* < 0.001
FC—1b (Total of whole grains)	17.12 ± 8.48	22.95 ± 9.17	F_(1, 209)_ = 22.12; *p* < 0.001
Muesli	14.89 ± 12.32	18.80 ± 14.00	F_(1, 207)_ = 3.91; *p* = 0.049
Whole grain bread	14.45 ± 8.54	18.99 ± 9.40	F_(1, 209)_ = 16.23; *p* < 0.001
Whole grain pasta	9.37 ± 8.11	11.22 ± 9.36	F_(1, 209)_ = 1.65; *p* = 0.201
Whole grain rice	5.87 ± 6.57	8.96 ± 8.26	F_(1, 209)_ = 7.17; *p* = 0.008
Other whole grains	6.07 ± 6.35	10.36 ± 9.18	F_(1, 209)_ = 12.03; *p* = 0.001
FC—2 (Total of beans and seeds)	28.47 ± 13.89	23.70 ± 13.74	F_(1, 209)_ = 7.12; *p* = 0.008
Nuts & seeds	22.25 ± 13.21	16.11 ± 12.67	F_(1, 209)_ = 13.04; *p* < 0.001
Legumes	15.98 ± 10.65	15.71 ± 10.74	F_(1, 209)_ = 0.23; *p* = 0.630
FC—3 (Total of fruit and vegetables)	34.09 ± 13.03	26.84 ± 11.77	F_(1, 209)_ = 19.30; *p* < 0.001
Vegetable juice	5.48 ± 9.74	5.70 ± 11.58	F_(1, 209)_ = 1.01; *p* = 0.315
Fruit	19.93 ± 9.30	18.16 ± 8.73	F_(1, 209)_ = 2.92; *p* = 0.089
Vegetables	34.73 ± 12.56	27.08 ± 10.50	F_(1, 209)_ = 22.01; *p* < 0.001
FC—4 (Total of dairy)	9.70 ± 12.11	10.77 ± 9.67	F_(1, 209)_ = 2.00; *p* = 0.159
Milk	7.57 ± 11.31	9.67 ± 11.71	F_(1, 209)_ = 3.00; *p* = 0.085
Cheese	7.10 ± 8.89	8.12 ± 8.05	F_(1, 209)_ = 1.76; *p* = 0.187
Yogurt	7.81 ± 11.00	7.17 ± 9.09	F_(1, 209)_ = 0.04; *p* = 0.833
FC—5: Dairy alternatives	18.08 ± 15.04	13.69 ± 15.51	F_(1, 209)_ = 6.44; *p* = 0.012
FC—6 (Total of meat)	4.95 ± 9.81	12.46 ± 13.70	F_(1, 209)_ = 19.26; *p* < 0.001
FC—6a (Total of unprocessed meat)	5.43 ± 10.68	13.04 ± 14.47	F_(1, 209)_ = 17.24; *p* < 0.001
Chicken	2.42 ± 5.16	4.98 ± 6.35	F_(1, 209)_ = 12.75; *p* < 0.001
Beef and pork and deer	4.34 ± 8.90	11.25 ± 13.20	F_(1, 209)_ = 18.29; *p* < 0.001
FC—6b (Total of processed meat)	3.93 ± 8.40	10.52 ± 12.67	F_(1, 209)_ = 19.72; *p* < 0.001
Fried nuggets	1.32 ± 3.19	2.62 ± 3.64	F_(1, 209)_ = 11.67; *p* = 0.001
Hamburger	0.43 ± 1.44	1.67 ± 3.10	F_(1, 209)_ = 12.15; *p* = 0.001
Sausage	0.25 ± 1.20	1.47 ± 3.14	F_(1, 209)_ = 14.23; *p* < 0.001
Kebab	0.34 ± 1.01	1.57 ± 2.78	F_(1, 209)_ = 15.49; *p* < 0.001
Other processed meat	4.05 ± 9.51	9.78 ± 13.02	F_(1, 209)_ = 14.40; *p* < 0.001
FC—7: Meat alternatives	5.99 ± 6.02	6.16 ± 7.44	F_(1, 209)_ = 0.48; *p* = 0.488
FC—8: Fish	3.80 ± 5.70	5.57 ± 6.90	F_(1, 209)_ = 4.60; *p* = 0.033
FC—9: Eggs	6.91 ± 8.65	9.16 ± 8.86	F_(1, 209)_ = 4.22; *p* = 0.041
FC—10 (Total of oils)	10.24 ± 10.66	15.49 ± 14.99	F_(1, 209)_ = 4.60; *p* = 0.033
Butter	4.50 ± 8.76	8.00 ± 13.53	F_(1, 209)_ = 0.88; *p* = 0.348
Margarine	5.92 ± 8.73	7.49 ± 11.36	F_(1, 209)_ = 0.13; *p* = 0.717
Other oils	4.95 ± 5.36	7.74 ± 7.50	F_(1, 209)_ = 5.71; *p* = 0.018
FC—11 (Total of snacks)	9.83 ± 6.67	11.81 ± 7.63	F_(1, 209)_ = 2.98; *p* = 0.086
Sweet snacks	9.77 ± 6.43	10.51 ± 6.78	F_(1, 209)_ = 0.43; *p* = 0.511
Salty snacks	5.22 ± 6.66	7.66 ± 7.67	F_(1, 207)_ = 6.13; *p* = 0.014
FC—12 (Total of water)	39.28 ± 22.17	29.92 ± 18.09	F_(1, 209)_ = 9.77; *p* = 0.002
Water	61.92 ± 28.33	56.16 ± 26.33	F_(1, 209)_ = 2.24; *p* = 0.136
Unsweetened tea	25.36 ± 17.63	16.52 ± 14.25	F_(1, 209)_ = 17.48; *p* < 0.001
FC—13: Beverages	14.19 ± 5.22	13.40 ± 4.57	F_(1, 209)_ = 0.88; *p* = 0.350
FC—14: Alcohol	2.75 ± 3.77	5.06 ± 5.64	F_(1, 209)_ = 13.04; *p* < 0.001
**Part B—Umbrella clusters**			
FC—15 (Total of protein)	39.60 ± 14.30	38.64 ± 13.81	F_(1, 209)_ = 0.28; *p* = 0.599
FC—15a (Total of plant protein)	35.23 ± 14.88	30.12 ± 13.94	F_(1, 209)_ = 6.40; *p* = 0.012
FC—15b (Total of animal protein)	12.80 ± 14.72	18.73 ± 14.98	F_(1, 209)_ = 9.04; *p* = 0.003
FC—16: Processed foods & free/added sugar	23.27 ± 12.62	30.25 ± 15.62	F_(1, 209)_ = 10.81; *p* = 0.001
FC—17: Free/added sugar	13.62 ± 8.60	16.19 ± 11.21	F_(1, 209)_ = 1.57; *p* = 0.212

Note. Data are presented as mean ± standard deviation. The values are based on a calculated index ranging from 0 to 100 (points; %), representing an integrated scale from the frequency of food consumption within the past four weeks and the amount of food intake. FC—food clusters. Statistical methods: Kruskal–Wallis tests (F-values).

**Table 4:** Regression results for age- and sex-based interactions in food clusters.

	Age			Sex *		
	β	95%-CI	*p*	β	95%-CI	*p*
FC—1a (Total of refined grains)	−0.07	[1.08, −1.21]	0.908	5.58	[8.03, 3.13]	<0.001
FC—1b (Total of whole grains)	−0.48	[0.66, −1.62]	0.407	6.01	[8.46, 3.56]	<0.001
FC—2 (Total of beans and seeds)	−0.39	[1.41, −2.19]	0.673	−4.63	[−0.77, −8.49]	0.019
FC—3 (Total of fruit and vegetables)	−2.11	[−0.51, −3.72]	0.010	−6.45	[−3.01, −9.89]	<0.001
FC—4 (Total of dairy)	0.43	[1.88, −1.02]	0.558	0.91	[4.02, −2.20]	0.565
FC—5 (Dairy alternatives)	−0.04	[1.95, −2.02]	0.971	−4.38	[−0.12, −8.64]	0.044
FC—6a (Total of unprocessed meat)	1.29	[2.90, −0.32]	0.115	7.13	[10.58, 3.67]	<0.001
FC—6b (Total of processed meat)	1.10	[2.45, −0.25]	0.110	6.18	[9.08, 3.28]	<0.001
FC—7 (Meat alternatives)	0.32	[1.19, −0.55]	0.470	0.05	[1.91, −1.81]	0.001
FC—8 (Fish)	0.49	[1.30, −0.32]	0.239	1.58	[3.32, −0.16]	0.074
FC—9 (Eggs)	1.09	[2.22, −0.04]	0.058	1.84	[4.26, −0.58]	0.136
FC—10 (Total of oils)	0.87	[2.52, −0.78]	0.299	4.92	[8.45, 1.38]	0.007
FC—11 (Total of snacks)	−0.11	[0.81, −1.04]	0.808	2.02	[4.00, 0.04]	0.046
FC—12 (Total of water)	−1.26	[1.41, −3.93]	0.355	−8.88	[−3.16, −14.61]	0.003
FC—13 (Beverages)	0.30	[0.94, −0.35]	0.366	−0.91	[0.48, −2.29]	0.198
FC—14 (Alcohol)	0.12	[0.72, −0.49]	0.703	2.27	[3.57, 0.97]	0.001
FC—15a (Plant protein)	−1.83	[0.05, −3.70]	0.056	−4.43	[−0.41, −8.44]	0.031
FC—15b (Animal protein)	1.48	[3.40, −0.44]	0.130	5.38	[9.50, 1.26]	0.011
FC—16 (Processed foods & free/added sugar)	−0.15	[1.67, −1.97]	0.872	7.03	[10.94, 3.13]	<0.001
FC—17 (Free/added sugar)	−0.14	[1.13, −1.42]	0.826	2.62	[5.36, −0.12]	0.061

Note. * The female sample is considered the reference. β—regression coefficient. CI—confidence interval. p—*p*-value. FC—food clusters. Statistical methods: Kruskal–Wallis tests (F-values).

## Data Availability

The datasets generated during and/or analyzed during the current study are not publicly available, but may be made available upon reasonable request. Participants will receive a brief summary of the results of the “NURMI Study”, if desired.
